# Comparative analysis of FKBP family protein: evaluation, structure, and function in mammals and *Drosophila melanogaster*

**DOI:** 10.1186/s12861-018-0167-3

**Published:** 2018-03-27

**Authors:** George Ghartey-Kwansah, Zhongguang Li, Rui Feng, Liyang Wang, Xin Zhou, Frederic Z. Chen, Meng Meng Xu, Odell Jones, Yulian Mu, Shawn Chen, Joseph Bryant, Williams B. Isaacs, Jianjie Ma, Xuehong Xu

**Affiliations:** 1 National Engineering Laboratory for Resource Development of Endangered Crude Drugs in Northwest of China, Xi’an, 710062 China; 2 Laboratory of Cell Biology, Genetics and Developmental Biology, Shaanxi Normal University College of Life Sciences, Xi’an, 710062 China; 3 Ohio State University College of Medicine, Columbus, OH USA; 4 Chen Wellness Clinics, Wichita, KS USA; 5 Department of Pharmacology, Duke University Medical Center, Durham, NC USA; 6 University of Maryland School of Medicine, Baltimore, MD USA; 7 State Key Laboratory for Animal Nutrition, Institute of Animal Science, Chinese Academy of Agricultural Sciences, Beijing, China; 8 Johns Hopkins University School of Medicine, Baltimore, MD USA

**Keywords:** FK506-binding protein, Tetratricopetide receptor, Transient receptor potential, Peptidyl-prolyl isomerase, Ryanodine receptor, Inositol 1, 4, 5-trisphosphate, Phospholipase C, Notch, Hsp90

## Abstract

**Background:**

FK506-binding proteins (FKBPs) have become the subject of considerable interest in several fields, leading to the identification of several cellular and molecular pathways in which FKBPs impact prenatal development and pathogenesis of many human diseases.

**Main body:**

This analysis revealed differences between how mammalian and *Drosophila* FKBPs mechanisms function in relation to the immunosuppressant drugs, FK506 and rapamycin. Differences that could be used to design insect-specific pesticides. (1) Molecular phylogenetic analysis of FKBP family proteins revealed that the eight known *Drosophila* FKBPs share homology with the human FKBP12. This indicates a close evolutionary relationship, and possible origination from a common ancestor. (2) The known FKBPs contain FK domains, that is, a prolyl cis/trans isomerase (PPIase) domain that mediates immune suppression through inhibition of calcineurin. The dFKBP59, CG4735/Shutdown, CG1847, and CG5482 have a Tetratricopeptide receptor domain at the C-terminus, which regulates transcription and protein transportation. (3) FKBP51 and FKBP52 (dFKBP59), along with Cyclophilin 40 and protein phosphatase 5, function as Hsp90 immunophilin co-chaperones within steroid receptor-Hsp90 heterocomplexes. These immunophilins are potential drug targets in pathways associated with normal physiology and may be used to treat a variety of steroid-based diseases by targeting exocytic/endocytic cycling and vesicular trafficking. (4) By associating with presinilin, a critical component of the Notch signaling pathway, FKBP14 is a downstream effector of Notch activation at the membrane. Meanwhile, Shutdown associates with transposons in the PIWI-interacting RNA pathway, playing a crucial role in both germ cells and ovarian somas. Mutations in or silencing of dFKBPs lead to early embryonic lethality in *Drosophila*. Therefore, further understanding the mechanisms of FK506 and rapamycin binding to immunophilin FKBPs in endocrine, cardiovascular, and neurological function in both mammals and *Drosophila* would provide prospects in generating unique, insect specific therapeutics targeting the above cellular signaling pathways.

**Conclusion:**

This review will evaluate the functional roles of FKBP family proteins, and systematically summarize the similarities and differences between FKBP proteins in *Drosophila* and Mammals. Specific therapeutics targeting cellular signaling pathways will also be discussed.

## Background

Since FK506-binding proteins (FKBP) were first identified, much progress has been made in understanding the mechanisms involved in regulating the immunological process, steroid signaling, and the resulting roles they play in physiological processes. Over the past years, FKBPs have emerged as a potential therapeutic target for a wide-range of endocrine- and cardiovascular-related diseases. As a result, several researches have focused on determination and development of novel therapeutic drugs that target FKBPs [1]. FKBPs are a highly conserved group of proteins known to bind to FK506, an immunosuppressive drug. Though FK506-FKBP complexes bind to calcineurin and inhibit its phosphatase activity, FKBP plays its immunosuppressive roles and inactivates the nuclear factor of activated T-cells [2–5].

FKBPs are named according to their molecular mass; the smallest members of FKBPs are composed almost entirely of a PPIase motif in a single FK domain, whereas larger FKBPs are composed of domains that are functionally independent. Thus, in addition to the peptidyl-prolyl isomerase (PPIase) motif, the *Drosophila* FKBP59 (FKBP52 and FKBP51 in mammals) have a Tetratricopeptide receptor (TPR) domain which functions independently by mediating protein-protein interactions. These TPRs have been identified in proteins involved in regulating transcription and protein transportation [3, 4, 6]. In spite of the growing evidence for the important roles of various FKBPs, knowledge of their specific functions remain obscure. Particularly, information on the role of FKBPs in the development of multicellular organisms is scanty [7].

The review will focus on the description and functional roles of the FKBP family members. Comprehensive analysis of these proteins in Mammals and *Drosophila melanogaster* indicates that many FKBPs and their functional complexes have potential therapeutic applications.

## Evolutionary and function linkage between *Drosophila* and mammal FK506-binding protein family

The immunophilins, including FKBP proteins, found in most organisms are involved in a range of biochemical processes such as transcription, protein folding, protein trafficking, and receptor signaling [2–4]. The number of immunophilins in different organisms differs greatly. In single cell organisms, *Escherichia coli* contains 6 immunophilins and the *Saccharomyces cerevisiae* (yeast) genome contains 4 FKBP proteins. In both *D. melanogaster* and *Caenorhabditis elegans* genomes, there are 8 FKBPs. In mammals such as *Mus musculus* (mouse) and *Homo sapiens* (human) genomes, 18 FKBPs have been documented up to date. In plants, both *Arabidopsis* and *Chlamydomonas* have 23 FKBPs while *Oryza sativa* contains 29 FKBPs [2, 7–11].

**Fig. 1:**
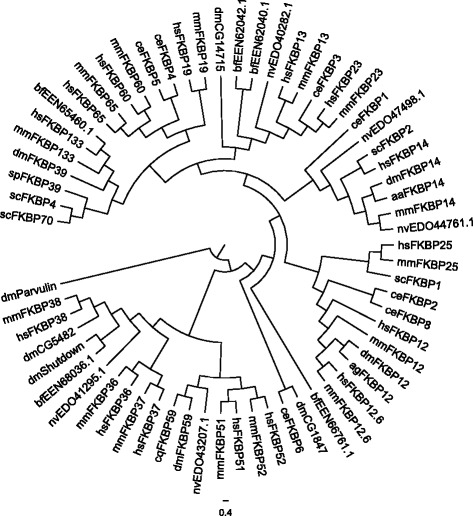
Molecular phylogenetic analysis of FKBP family proteins by Maximum Likelihood. The evolutionary tree is presented to compare each subgroup with family members present in other species. The evolutionary history was inferred using the Neighbor-Join and BioNJ algorithms. The percentage of replicate trees in which the associated taxa clustered together in the bootstrap test (500 replicates) is shown next to the branches. Evolutionary analyses were conducted in MEGA7. The proteins were analyzed as intact sequences. The analysis involved genes from *C. elegans*(ce), *Schizosaccharomyces pombe* (sp), *Arabidopsis thaliana* (at), *Anopheles gambia* (ag), *Aedes albopictus* (aa), *Culex quinquefasciatus* (cq), *Drosophila melanogaster* (dm), *Mus musculus* (mm) and *Homo sapiens* (hs)

Molecular phylogenetic analysis of FKBP family proteins revealed that *Drosophila* FKBPs form a close association with other organisms (Fig. 1). This analysis demonstrates that the eight known FKBPs in *Drosophila* share homology with the human FKBP12. This indicates that they have a much closer evolutionary relationship and might have originated from a common ancestor.

**Fig. 2:**
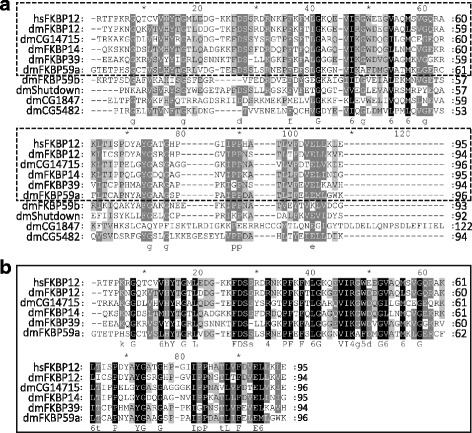
Multi-alignment analysis of FKBP family proteins. A multiple sequence alignment of domains from (**a**) overall FKBP family proteins and (**b**) only the well-established FKBPs of *Drosophila melanogaster* show relative similarities among FKBP12 paralogues and orthologues at the amino acid level (black). The dotted box in A indicates domains of well-established FKBP proteins among the overall FKBP family proteins

The phylogenic tree divides FKBPs into two groups: those with a TPR domain and those without a TPR domain. The FKBPs without TPR are further divided into three subgroups. Members of the first sub-group have a single FKBP domain localized in the cytoplasm (for example, FKBPP12). Members of the second group have one to four FKBP domains. They also have with diverse motifs such as EF-hand and calcium binding, and ER signaling peptide localized in the ER (this includes FKBP14). Members of the third sub-group consist entirely of one FKBP domain with an additional motif, and are localized in the nucleus (for example, FKBP39). The FKBPs with TPR including FKBP59 are localized in the cytoplasm, ER, mitochondria or nucleus. Our multiple-sequence alignment analysis exhibited a highly conserved FKBP domain among some FKBP families (Fig. 2a and b), which reiterates that the FKBP genes underwent their functional differentiation with the coding for diverse roles of these proteins.

Furthermore, based on their functional domains, *Drosophila* FKBPs can be grouped into two categories. The first category, represented by the FKBP12 and CG14715 (FKBP13), consists principally of FKBP domain. In the second category, this FKBP family contains an additional domain such as the EF-hand domain, cwf/cwc superfamily domain, or TPR domain. Category 2 members include FKBP14, FKBP39, Shutdown (CG4735), CG1847, CG5482, and FKBP59. Among these members, the FKBP14 contains the additional EF-hand domain known to bind to Ca^2+^ [12, 13]. FKBP39 contains the cwf/cwc superfamily domain, which is a complex of Cef1 and may be implicated in pre-RNA splicing, through spliceosome [14, 15]. The remaining FKBPs are those with a TPR domain in addition to the FKBP domain. The TPR domain is believed to mediate protein–protein interactions, specifically by serving as binding sites for ubiquitous and abundant molecular chaperone, heat shock protein 90 (Hsp90). Although the discovery of FKBPs originally centered on their interplay with the FK506 and rapamycin ligands, FKBPs are now understood to have complex and varied functions in their natural environment [16, 17].

In the second category, three *Drosophila* FKBPs—CG1847, CG5482, and FKBP59—are annotated to share the domain architecture of CG4735/FKBP6, in which FKBP domains are followed by a TPR domain. *Shut-down* encodes a novel *Drosophila* protein similar to the heat-shock protein-binding immunophilins and is a potential fourth member of this group. Though its TPR domain has substantially diverged in comparison to the others, its secondary structure predicts the putative TPR. The *shut-down* gene is essential for the normal function of the germline stem cells. Analysis of weak loss-of-function alleles has confirmed that shut-down is also required at later stages of oogenesis [18, 19]. Apart from the shutdown, the dFKBP12 gene is also expressed in follicle cells during oogenesis, while dFKBP14, dFKBP39, and dFKBP59 are expressed throughout the *Drosophila* life-cycle [7, 20–22].

Noticeably, in *Drosophila*, the FKBP domain is a signature structure in every FKBP gene. Except for CG1487 gene, the FKBP domain ranges from 92 to 96 amino acids in every FKBP as summarized in Tables 1 and 2, and Fig. 2a and b. However, aside from the FKBP domain, the other domain might provide more information on predicting their further function.

**Table 1:** FKBP nomenclature in *Drosophila*

Gene symbol/Name	Exon	Location	FKBP domain (AA)	Transcripts (bp)	CDS (bp)
CG11001/FKBP12	4	Chromosome 2R: 19290109–19,290,962	94	633	327
CG14715/FKBP13	2	Chromosome 3R: 11674221–11,674,923	96	640	417
CG9847/FKBP14	9	Chromosome 2R: 21487167–21,497,929	95	1311/1350*	651
CG6226/FKBP39	3	Chromosome 3R: 15331584–15,333,133	94	1299	1074
CG4535/FKBP59	9	Chromosome 2 L: 9888005–9,890,119	96/93*	1494/1587*	1320
CG4735/Shutdown	3	Chromosome 2R: 23841461–23,843,286	92	1694	1826
CG1847	3	Chromosome X: 11869170–11,871,168	122	1776	963
CG5482	9	Chromosome 2R: 18629594–18,632,045	94	1780/1958*	1194

*If there are more than two transcripts or two coded proteins, the two largest are listed

**Table 2:**
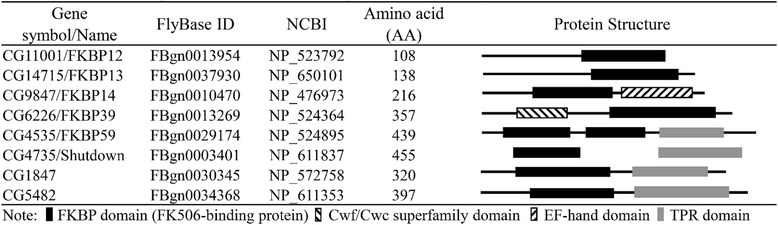
Schematic representation of the Drosophila FKBP family members

**Fig. 3:**
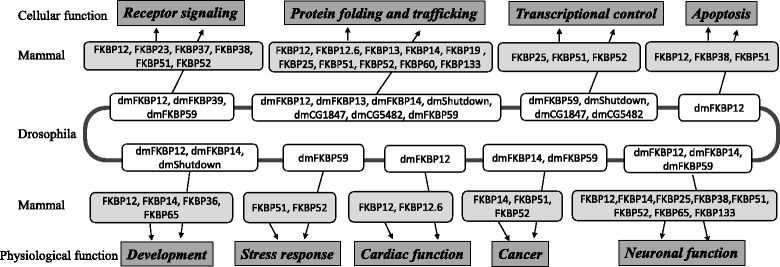
Summary of FKBPs’ involvement in different biological processes in Mammal and *Drosophila*. The cellular functions of the various FKBPs listed on the top include receptor signaling, protein folding and trafficking, transcriptional control, and apoptosis. The FKBPs are also associated with different physiological roles within stress response, cardiac function, cancer genesis, neuronal function, and during animal development

Furthermore, sequence identity reveals greater intimacy in protein structure, function maintenance, and differentiation during the evolution of both mammals and insects. *Drosophila* FKBP, CG14715 (dFKBP13) shares 57% sequence identity with mammalian FKBP13 implicating their involvement in intracellular transport and protein folding, a prolyl-isomerase thought to function as an ER chaperone [7]. Within *Drosophila,* a strong homology of 43% sequence identity between FKBP12 and FKBP14 suggests that they may share similar biological roles such as their PPIase activity. However, there is a lack of detailed evidence. Spatiotemporally, FKBP12 and FKBP14 are distributed unambiguously into different fractions: the FKBP12 located almost entirely in the cytosol, whereas FKBP14 is assembled (resides) exclusively in the rough endoplasmic reticulum fraction. As the broadly expressed ER resident protein, FKBP14 is detected throughout *Drosophila* embryo suggesting its critical role in the development. FKBP14 Null mutations are lethal throughout the larval and pupal stages of the development, and the survivor exhibits defects in the eye, wing and sensory bristle development. Consequently, the mutants do not appear to induce the unfolded protein response, which indicates that FKBP14 is associated with the dysregulation of Notch signaling. Additionally, the expression levels of CG1847 in sensory organs and precursors are 2-fold more than its expression levels in epithelial cells. This implies that CG1847 could be involved in cell cycle-associated transcription and istargeted on both mitosis and sensory physiology [7, 23, 24]. Furthermore, CG5482 is reported to be a Myc target gene and its expression is directly regulated by dMyc (*Drosophila* Myc) in larva [25, 26]. Furthermore, c-MYC regulates over 15% of genomic genes through binding to the consensus sequence—termed E-boxes in mammal cells. It is therefore possible that CG5482 plays a role in intracellular signaling pathways. The functional involvements of FKBPs in biological process are summarized in Fig. 3. The cellular and physiological processes are two categories of FKBPs’ roles in Mammal and *Drosophila*. FKBPs can target on various pathways in cells via interaction with components of receptor signaling, protein folding and trafficking, transcriptional control and apoptosis, whereas the physiological functions are associated with embryonic development, stress response, cardiac function, cancer tumorigenesis and neuronal function.

## FKBPs and critical cellular signalling pathways

### FKBP, TRP/TRPL, and RyR in calcium signaling pathway

Genetic analysis in *Drosophila* identified Transient Receptor Potential (TRP) as a potential store-operated channel, which are essential for the light-induced cation conductance in photoreceptor cells. This cytoplasmic TRP in the configuration of a proposed supramolecular complex is important for feedback regulation and/or activation associated with rhodopsin, phosphor-lipase C, protein kinase C, calmodulin, and the PDZ domain-containing protein [27, 28]. Involved in the inositol 1, 4, 5-trisphosphate signaling pathway for photo-transduction, the TRP appears as a putative Ca^2+^ entry channel present in fly photoreceptors. The interest in *Drosophila* photo-transduction originates from the apparent similarities in signaling in fly photoreceptor cells and the wide range of excitable and non-excitable cells in mammals that rely upon phosphoinositide signaling and conclude with the opening of Ca^2+^-permeable channels. However, until recently, the identities of the Ca^2+^ influx channels and the mode of activation have been elusive [27, 29, 30].Thus, In the *Drosophila* eye, the Ca^2+^ − permeable TRP and Transient Receptor Potential-like (TRPL) channels activation by photo-stimulation of rhodopsin leads to a response which depends on phospholipase C This results in depolarization of the photoreceptor cell followed by Ca^2+^-mediated feedback regulation of the visual signaling cascade. The biochemical or biophysical link between stimulation of a phospholipase C and channel activation remains vague [29, 30].

The effect of immunophilin ligands on neuronal survival and growth may involve Transient Receptor Potential Canonical (TRPC) channel function since the neurotrophic action of FK506 appears to be arbitrated by FKBP52 and by extension FKBP59 in *Drosophila.* A recent study shows that the binding of FKBP52 in the proline-rich region at the C-terminus of TRPC1 may prevent homer interaction among the N-terminal site of TRPC1. The mutation of Pro to Gln at the second LP dipeptide in *Drosophila* TRPL channels is able to eliminate the binding of FKBP59 [31, 32]. A fascinating feature of TRPL shared by the trimeric G protein and the arrestin is the dramatic light-dependent translocation. This exhibits as the sustained light response in wild-type flies and transient light response in the *trp* mutant [27, 29]. Based on sequence homology, the mammalian TRPC family consists of seven orthologs which can be divided into two major sub-groups. The first sub-group is composed of TRPC1, -C4, and -C5. The second sub-goup is composed of TRPC3, -C6, and -C7. TRPC2 is pseudogenic. *Drosophila* TRP or TRPL is about 40% identical to the TRPC1 and TRPC4. Intriguingly, the putative FKBP binding domain found in *Drosophila* TRPL is found in all the mammalian TRPC proteins as well [31, 33, 34].

In mammals, it has been broadly accepted that FKBP12 and FKBP12.6 are the most important regulators of the RyR ion channel’s interactions with other proteins such as Ca_v_, FKBPs, and calmodulin. The association of FKBP 12 and FKBP12.6 with RyR1 and RyR2 are crucial and well-studied for Excitation-Constriction (E-C) coupling in both skeletal and cardiac muscles as well as in the brain [3, 12, 29]. The binding of Ca^2+^ to the EF-hand domain of FKBP12 or FKBP12.6 facilitates the transmembrane helices to form a pore and increases the plausibility of gate opening with large-amplitude collective motion. The suggested mechanism also explains the effect on gating induced by the binding of regulators to the cytoplasmic domain of RyR. Furthermore, several studies have confirmed in *Drosophila* that the FKBP12’s potential function in E-C coupling in the muscles and brain involve RyR’s function in excitable and non-excitable cells [12].

### FKBPs functionally associates with notch signaling pathway

The conserved part of Notch signaling pathway is composed of ligands (Delta and Jagged), Notch receptors, and the transcription factors. The Notch signaling pathway regulates a wide spectrum of fundamental processes and different cellular programs such as proliferation, apoptosis, migration, growth and differentiation. Only recently has the pathway been appreciated for its role in the maintenance of adult tissues during postnatal life and abnormal activation in the pathogenesis of human diseases such as cancer. Despite this enormous breakthrough, the mechanism of Notch signaling activation in solid tumors is yet to be discovered [35, 36]. The involvement of FKBPs in this signaling pathway provides a new prospect for understanding the molecular mechanism of the related diseases.

In *Drosophila*, FKBP14 is known to be associated with the dysregulation of Notch signaling through downstream targeting and is required for metazoan development. Albeit trafficking of Notch to the membrane appears unaffected in the FKBP14 mutant, the expression levels of several Notch components including Cut, Wingless and Enhancer of split are reduced significantly. Correspondingly, PSN (presenilin-1 or presenilin-2) protein levels and γ-secretase activity are reduced in FKBP14 null mutants as well. This phenomenon suggests that the targeting site of FKBP14 could be downstream of the Notch activation at the membrane [7, 35]. Taken together, FKBP14 is an essential gene that regulates Presenilin protein levels and Notch signaling in *Drosophila*.

### *Shutdown* via piRNA pathway highly affects generation of reproductive cells

Shutdown, a member of the FKBP immunophilin family, was recently discovered to be associated with transposons in the PIWI-interacting RNA (piRNA) pathway, which plays a crucial role in both germ cells and somatic ovarian cells [37]. Transposon have a significant effect on genomic instability and even low level expression can drive evolution in the long term. By suppressing the activity of transposable elements (TE), the piRNA pathway protects genome integrity during germ stem cell (GSC) differentiation and oocyte development. This piRNA silencing of transposons in reproductive tissues thereby significantly reducing the frequency of spontaneous mutations in progeny [15, 38–43]. PiRNAs also play a role in the somatic cells encapsulating and supporting the growth and maturation of germ cells. These stromal cell piRNAs shape piRNA populations within germ cells by targeting mobile elements via a ping-pong cycle and shaping germ line transposon mRNAs [44–47].

*Shu* (Shutdown/CG4735) was first recognized as a female sterile gene in *Drosophila.* It comprises of an evolutionarily conserved co-chaperone which contains a PPIase motif and a TPR domain for an FKBP family proteins (Table 2) [15, 18]. Acting downstream of the piRNA pathway, Shutdown is known to be a part of both the nuage and Yb bodies [37]. These nuage and Yb bodies are thought-out to be pivots of piRNA biogenesis [15, 37]. It is established that mutations in *Shu* disturbs germ cell division and ultimately cause the germline stem cells to fail entirely [15]. Two robust alleles caused sterility in both males and females, but a third allele with mutation did not alter male fertility. In mutant females, stem cells that strongly divide also produced defective egg chambers that ceased mid-oogenesis [15, 48, 49]. Analyzing the expression patterns of transposon and small RNA libraries in Shu knockdown cells and deficient animals suggest the important role Shutdown play in piRNA biogenesis or its complex stabilization [15]. This critical function could be performed through the loading of piRNAs into PIWI-family proteins [15]. As Shutdown constituent domains have PPIase characteristics and the capacity to collaborate with the HSP90 family of chaperone proteins, any of these activities could determine the role of Shutdown in the piRNA pathway [15, 49].

The association between Shutdown and Hsp90 potentially implicates the chaperone system in secondary piRNA biogenesis [50]. However, treatment of BmN4 cells with FK-506 failed to have any overt effect on piRNA biogenesis [50]. Thus, although available evidence indicates a link between Shutdown and Hsp90, it is yet to be seen if the definite mechanisms of action are conserved between insects and mammals [15, 50]. Obviously, understanding the function of Shutdown in either ping-pong cycle or primary biogenesis will require further genetic manipulation to reiterate the aspects of the piRNA pathway in vitro [15].

## FKBPs and human diseases

### FKBPs regulate tumorigenesis associated with steroid hormone receptors

In mammals, FKBP51 and FKBP52 (dFKBP59), protein phosphatase 5 (PP5), and Cyclophilin 40 (CyP40) have been reported to couple with Hsp90 within steroid receptor (SHR)-Hsp90 hetero-complexes [3, 6, 51]. Interestingly, the expression of two co-factors, FKBP51 and FKBP52, which inhibit and facilitate Glucocorticoid receptor nuclear translocation, respectively, were also negatively correlated. This suggests that these co-factors are not coordinately up-regulated to ‘balance’ their effects, but may be reciprocally regulated to exaggerate their individual effects [51]. Since *Drosophila* only has FKBP59, it is important to determine how it plays such role. Their well-known effects on steroid receptors make these immunophilins potential drug targets in the pathways associated with normal physiology and a variety of steroid-based diseases including prostate cancer, breast cancer, stress-related diseases, and metabolic diseases [3, 6, 52–55].

To date, researchers have focused their efforts on the identification and development of novel drugs targeting FKBP51 and FKBP52 primarily by utilizing their functional domains. FKBP51 and FKBP52 share 70% similarity and have 2 PPIase (FK1 and FK2) domains, respectively. Currently, the FK2 domain is enigmatic. This domain is structurally similar to the FK1, but lacks PPIase activity as there is no binding to the immunosupressant FK506. Deletion of three amino acids (D195, H196, and D197) in the FKBP51 FK2 domain does not interrupt Hsp90 binding but rather affects the integration of the protein into progesterone receptor complexes [1, 6, 55–58]. A reasonable explanation is that the deletion of these three amino acids reduces the interaction of FK2 domain with other components of the receptor complex including the receptor itself. The interaction of TPR domain with the SHR complexes of Hsp90 facilitates the binding of Hsp90 to the FKBPs [1, 6, 16].

FKBP52 FK1 is the functional domain of FKBPs while the rest of the protein provides the interaction platform from which FKBP52 associates directly with steroid receptor-chaperone complexes [1, 16]. The FKBP52 FK1 domain, stabilized by the p23 co-chaperone, interacts with the Hsp90-receptor complex to bind with the receptor ligand binding domain and directly affect the hormone binding affinity of steroid receptor-chaperone receptors [1, 58]. Interruption of this interaction between FKBPs and receptor complex proteins suppresses SHR retro-transport at level comparable to the presence of Hsp90 inhibitors [1, 6, 16]. This implies FKBPs as a key component of steroid hormone receptor function, inhibition of which could be as potent as Hsp90 inhibition in steroid hormone receptor associated cancers.

In mammals, FKBP51 and FKBP52 play significant roles in embryonic development [1]. It has been proposed that FKBP51 and FKBP52 (dFKBP59) might regulate transcription at the site of gene promoters but more evidence is needed in both mammals and *Drosophila.*

There has been growing interest in the link between FKBPs and cancer. This is because of up-regulated activity of mTOR (mammalian target of rapamycin) by FKBPs, especially in cells lacking functional PTEN, subverting protein synthesis and tumor growth, The successful treatment of cancer using immunosuppressants FK506 and rapamycin, highlights the potential of targeting FKBPs in cancer therapy. In leukemia, inhibiting FKBP51 by rapamycin-abrogated doxorubicin-induced activation of NF-kB enhanced drug-induced apoptosis [1, 57–60].

The FKBP51 protein plays important roles in the regulation of a variety of signaling pathways while its expression level changes in different conditions. By regulating steroid receptor maturation, the Akt and nuclear factor κB (NF-κB) signaling pathways, FKBP51 is implicated in tumorigenesis and in the response to chemotherapy [6, 55, 61]. Indeed, either decreasing of AKT-S473 phosphorylation or enhancing sensitivity to Gemcitabine due to the over-expression of FKBP51 is dependent on the AKT-S473 phosphatase PHLPP (PH domain and Leucine-rich repeat Protein Phosphatases). Interestingly, FKBP51 affiliates with AKT through its conserved FKBP domains and interacts with PHLPP through a C-terminal TPR domain. However, the PPIase activity of FKBP51 is not required for its effects on AKT phosphorylation and does not appear to affect PHLPP activity either. Collectively, several reports demonstrated that FKBP51 served as a scaffold to facilitate the PHLPP-mediated dephosphorylation of AKT-S473. In silenced FKBP51 cells, irradiation led to apoptosis rather than autophagy as induced in control cells. Prevention of apoptosis in control cells involved FKBP51-dependent initiation of NF-κB upon irradiation [6, 55, 62].

Overall, it appears that depending on cell and cancer type, promoting or reducing FKBP51 activity may produce a beneficial effect against cell proliferation of cancer cells. Most likely, different aspects of FKBP51 were involved for its divergent actions, which could eventually be exploited for drug development for specific diseases [1, 62, 63].

### Potential role of FKBPs in Alzheimer’s disease

Alzheimer’s disease (AD) is a tau pathology which includes the collection of tau proteins into neurofibrillary tangles (NFTs) [53]. Post-translational modifications affect the conformation of the tau protein, which is inherently a disorganized protein [53]. Several studies have reported AD as a progressive neurodegenerative disorder which is the most common form of dementia associated with age [53, 64, 65]. Although there is evidence of the interaction between FKBP12 and Amyloid Precursor Protein (APP) [53, 65], a physiological function of the interaction has not been proposed yet. However, the FKBP12/APP interplay is suspected to have a role in the amyloidogenic processing of APP, thereby affecting the expression level of Aβ peptides [65]. This will reverberate the function of Pin1 (protein interacting with NIMA-1) on Alzheimer APP [65]. The FKBP52 protein is adequately expressed in the nervous system, with elevated expression in both damaged and degenerating brain regions [64]. After several fundamental observations, critical data have focused on the pathogenesis of FKBP52 and AD. It would be reasonable to speculate that FKBPs with PPIase motif may configure to Tau structure and affect Tau function. This is because, PPIases are known to modulate protein phosphorylation [65]. Whether FKBP12 interaction with the APP-intracellular domain impact on Aβ production or whether its exposure to FK506 would have an advantageous or adverse effect is unclear [17, 53, 65, 66]. However, it is obvious that FKBP12 not only exhibits its co-localization with NFTs to prevent Tau oligomerization, but also inhibit the in vitro Tau aggregation [65].

Recent studies show that Hsp90 co-chaperones regulate Tau stability, aggregation, and clearance. Thus, signifying additional functional role of FKBPs in AD-related development [65, 67]. Once FK506/rapamycin derivatives are capable of modulating PPIase activity of FKBP51 and FKBP52, they may offer a unique approach to prevent or minimize the pathogenic effects of misfolded Tau [6, 53, 63]. Efforts are currently underway to pharmacologically target the FKBP proteins. The outcome of which will enhance our understanding of the interaction between FKBP and receptor-chaperone complex [1, 68]. These co-chaperones further suggest that FKBP51 and FKBP52 act on both Aβ metabolism and toxicity [64, 68]. The transgenic *D. melanogaster* strains over-expressing human Aβ 42 peptides present degeneration of the nervous system with concomitant learning and memory defects, in a dose-dependent manner [17, 64, 66]. Mutations in the *Drosophila* FKBP59 (mammalian FKBP52) exacerbate Aβ toxicity while transgenic flies that over-express wild type dFKBP59 decrease Aβ toxicity. The effects on Aβ phenotypes correspond with altered levels of the peptide suggesting that dFKBP59 may affect Aβ turnover in the above physiological processes. Therefore, the protective effect of dFKBP59 on Aβ toxicity during *Drosophila* aging was evident from the observations that dFKBP59 loss of function mutations potentiated Aβ toxicity, while over-expression of dFKBP59 delayed Aβ-induced phenotypes [17, 64, 66].

In human patient samples, the limited data has provided valuable information on the involvement of FKBP52 in Alzheimer disease. A comparison of FKBP52 protein expression in in both areas of the brains of people who had died of AD with those who had died with other non-AD neurological diseases showed that the AD samples had lower expression of FKBP52 compared to controls [17, 64, 68]. As the FK506-binding domain of FKBP52 is involved in APP binding, it is conceivable that immunophilin ligands such as FK506 and rapamycin may associate with Alzheimer disease as well. Furthermore, the role of FKBP51 in regulating Tau biology has also been well characterized. The over-expression of FKBP51 preserves Tau, while knock-down of FKBP51 leads to Tau degradation [17, 64, 65]. Thus, further studies will provide evidence to the role of ligands in modulating the toxicity of Aβ and may open the field for the development of a novel group of agents against AD.

## FKBPs are potential targets for potent insecticides

### Pharmacological mechanism of the immunosuppressant molecule suggests strategy for developing insect specific pesticide

In mammals, FK506 binds to all proteins in FKBP family by targeting on the surface within their deep cleft where the ligand is bound with hydrophobicity [69]. Tacrolimus, an immunosuppressant molecule used for treating patients who suffer from autoimmune disorders after organ transplant, was originally modified from FK506. In humans, the FKBP12-tacrolimus complex regulates the function of the calcineurin combined with the ciclosporin-cyclophilin complex by blocking transduction pathway in the T-lymphocyte [69]. Additionally, FKBP12 controls the EC-coupling function targeted on the gating of RyR in cardiomyocytes through engaging calcium-induced-calcium-releasing [70, 71]. Lack of FKBP12 or FKBP12.6 leads to sever cardiac dysfunction, and results in early death of *FKBP12* gene deletion mouse shortly after birth [72, 73]. The binding alternation of FKBPs to RyR on calcium signaling pathway provides the molecular mechanism of the death [74]. Recently, the crystal structure analysis on human FKBP25 in complex with FK506 revealed a FK506 binding domain (FKBD) at 1.8 Å resolution. The comparison between this complex and its rapamycin complex pointed out the microheterogeneity with strong interaction between rapamycin and the FKBP but a lower affinity between FK506 and the FKBP [75]. The characteristics of this pharmaceutics mechanism may highlight a direction for developing insect specific targeting drugs.

Binding affinity of the immunosuppressant molecule FK506 and rapamycin to their targeted protein determines the drug effects on their pharmaceutical function and is dependent on the number of hydrogen bonds between molecules. Per the crystal structure of the FK506 and hFKBP25 complex, four hydrogen bonds are responsible for the stabilization of the drug, which are between the backbone atoms of Lys170/Ile172 and the O2/O10 of FK506, as well as side-chain oxygen atoms of Asp146/Tyr198 and O6/O3 of FK506 [75]. In addition to these four hydrogen bonds, while in the rapamycin-hFKBD25 complex, there are more hydrogen bonds between atoms of Lys170 NZ/Lys170 NZ/Gly169 and oxygen at O8/O9/O13. This elucidates a stronger affinity of rapamycin to hFKB2 than that of FK506. Furthermore, the FK506 and hFKBD25 complex presents some extra nonbonded contacts at C12/C35/C36/C43/C45 of Ala206/ Ala206, Ile208/ Leu162/ Gln203/ Tyr198, which should be the consequences of the hydrogen bond association within the nuclear immunophilin bound to rapamycin [75, 76].

According to the knowledge gained from mammals, when developing insect-specific pesticide, the strategy should be based on the following categories. The characteristics of dFKBPs should be systematically analyzed within their FKBD and with focus on possible extra hydrogen bonds with the proteins. Additionally, building a small molecule library composed of chemical derivatives of two immunosuppressant drugs, rapamycin and FK506, could be another conceivable approach to the pesticide or insecticide.

### Comparison of FKBPs in *Drosophila* and mammals reveals potential targets for specific pesticides

It has become increasingly obvious that the Hsp90-binding PPIases play important physiological and potentially pathological roles in *Drosophila*. Elucidation of these roles, definition of the underlying molecular mechanisms, and identification of specific inhibitors will likely improve in the coming years and lead to the therapeutic targeting of individual PPIases that are specific to *Drosophila* or insects. Thus, the FKBPs that are unique to *Drosophila* or insects are the best target among this group of immunophilins.

*Drosophila* FKBP39 is a known nucleolar protein that interacts with both nucleosomes and the small subunit processome [77]. It has been shown that FKBP39 co-purifies with several kinetochore proteins [78] and associates with microtubules [79]. Several avenues have been investigated in a quest to identify an enzymatic activity. The most promising is the finding that the FKBP39 nucleoplasmin (NPL) domain binds divalent metal cations [77]. Insect growth and development are controlled by 20-hydroxyecdysone and juvenile hormone 1, the two main lipophilic hormones [80]. Interestingly, a recent report has suggested that two proteins, the 39-kDa FK506-binding protein (FKBP39) and the 21-kDa calponin-like protein (Chd64),are major components of a dynamic, multiprotein complex that crosslinks these two hormonal signaling pathways. This putative complex can bind to the juvenile hormone response element [81]. Intriguingly, the molecular basis of the interactions between FKBP39, Chd64 and the other components of the multiprotein complex is not understood. Recently, Kozlowska and colleagues [82] showed that the structure of Chd64 possesses a dual nature, consisting of a globular domain flanked by intrinsically disordered tails. Current studies indicate an unusual quaternary organization of FKBP39 that distinguishes this protein from other family members. The extreme plasticity and flexibility of the FKBP39 molecule, which may exist in some conditions in an alternative quaternary structure, may lay a foundation for this multifunctionality. Currently, the main function of FKBP39 is unknown. One very important feature of *Drosophila* FKBP39 is its central highly charged region, which is formed by acidic and basic stretches located between the NPL and FK-506 binding protein domains that lack stable structures [80].

Since transposons have a meaningful effect on genomic instability, the loss of control over mobile elements in any individual can threaten reproductive achievement. Therefore the piRNA pathway in *Drosophila* is essential in early oogenesis, suppressing the activity of TEs and safeguarding genome integrity during GSC differentiation and oocyte development [15, 38, 44]. Mutations in *Shu* disrupt germ cell division and ultimately cause the germline stem cells to fail entirely. Clearly, understanding the function of Shutdown in both primary biogenesis and the ping-pong cycle will require further studies to envisage its possibility as a drug target. The *Drosophila* RyR shares only 45% sequence homology with the mammalian isoforms of RyRs. There are regions of divergence between the mammalian and insect isoforms of RyRs, which could serve as potential targets for the potent insecticides that interact specifically with the insect but not the mammalian isoforms of RyR [83]. Similarly, the divergence that exists between insectoid and mammalian FKBPs could be a potential target for insecticides (Fig. 2a and b).

The observation that mutations in the *Drosophila* FKBP59 exacerbate Aβ toxicity suggests a significant role in Aβ turnover [17, 64, 66]. Also, since FKBP59 is implicated in light-induced conductance change in photoreceptor cells, it remains a potential target for therapeutics. Furthermore, the unique FKBPs in *Drosophila* and insects in general could also be explored for similar therapeutic purposes. Taken together, *Drosophila* continues to serve as a useful model for drug targeting and life enhancement research for both medical and agricultural purposes.

## Conclusion

The immunophilins, such as FKBPs, are highly conserved ubiquitous proteins and are engaged in a number of biochemical processes involving physiological activity and pathogenesis in both vertebrates and invertebrates. The fact that FKBP proteins are expressed ubiquitously but relatively in high levels of expression in the nervous system may implicate its involvement in endocrine and neurological function in human and *Drosophila*. FKBP52 (FKBP59) plays a crucial role in receptor translocation to the nucleus, and possibly regulate the receptors in the nucleus associated with Hsp90 and TRP/TRPL, thus regulating Tau stability, aggregation, and clearance in Alzheimer syndrome related processes.

Many other functions have been discovered in animals. In *Drosophila* eyes, FKBP59 associated with the Ca^2+^-permeable TRP and TRPL channels activation by photo-stimulation of rhodopsin functions for light-induced conductance change in *Drosophila* photoreceptor cells. In mammals, it has been suggested that FKBP 12 and FKBP12.6 are the most important regulators of RyR-related EC coupling in bones, cardiac muscle, and the brain. Moreover, FKBP51 and FKBP52 (dFKBP59), PP5, and CyP40 coupling with Hsp90 within steroid receptor-Hsp90 hetero-complexes are involved in embryonic development. FKBPs are also connected to cancer. Specifically, FKBP51 is associated with the mTOR pathway to enhance the activation of NF-κB and increase drug-induced apoptosis in leukemia. FKBP51 also plays a critical role in tumorigenesis by regulating steroid receptor maturation, the Akt, and NF-κB signaling pathways. Furthermore, FKBPs may play a role in neurodegeneration disease. Their intrinsic PPIase activity may provide a unique set of potential drug targets that can structurally modify and regulate phosphorylation of the Pro-rich proteins like Tau. Additionally, FKBPs are involved in a number of signal pathways, with FKBP14 and *Shu* implicated in Notch and piRNA pathways respectively.

Since most FKBPs have been involved in a variety of diseases and many served as therapeutic targets for these diseases, it is intriguing to explore the possibility of utilizing these characteristics in *Drosophila* and other organisms, in order to develop a disease as well insect-specific as drug for therapeutic purposes. The extensive research over the past decades witnessed a rapid expansion of our knowledge on the mechanism of the FKBP machinery. A combination of experimental approaches enabled a detailed understanding of the functional roles of the FKBPs. Thus, the comprehensive study of the FKBP machinery will not only contribute to the understanding of cellular protein folding mechanisms but also to the treatment of diseases and life enhancement.

## Abbreviations

AD: Alzheimer’s disease
APP: Amyloid Precursor Protein
Chd64: 21-kDa calponin-like protein
CyP40: Cyclophilin 40
E-C: Excitation-Constriction
FKBP: FK506-binding protein
GSC: Germ stem cell
Hsp90: Heat shock protein 90
mTOR: mammalian target of rapamycin
NPL: Nucleoplasm
Pin1: Protein interacting with NIMA-1
piRNA: PIWI-interacting RNA
PP5: Protein phosphatase 5
PPIase: Prolyl cis/trans isomerase
PSN: Presenilin
SHR: Steroid hormone receptor
TE: Transposable element
TPR: Tetratricopeptide repeat
TRP: Transient Receptor Potential
TRPC: Transient Receptor Potential Canonical
TRPL: Transient Receptor Potential-like

## References

[1] Storer CL, Dickey CA, Galigniana MD, Rein T, Cox MB (2011). FKBP51 and FKBP52 in signaling and disease. Trends Endocrinol Metab.

[2] Ahn JC, Kim D-W, You YN, Seok MS, Park JM, Hwang H (2010). Classification of rice (Oryza sativa l. japonica nipponbare) immunophilins (fkbps, cyps) and expression patterns under water stress. BMC Plant Biol.

[3] Lanner JT, Georgiou DK, Joshi AD, Hamilton SL (2010). Ryanodine receptors: structure, expression, molecular details, and function in calcium release. Cold Spring Harb Perspect Biol.

[4] Prakash A, Shin J, Rajan S, Yoon HS (2016). Structural basis of nucleic acid recognition by FK506-binding protein 25 (FKBP25), a nuclear immunophilin. Nucleic Acids Res.

[5] Stocki P, Sawickia M, Mays CE, Hong SJ, Chapman DC, Westaway D (2016). Inhibition of the FKBP family of peptidyl-prolyl isomerases induces abortive translocation and degradation of the cellular prion protein. MBoC.

[6] Li L, Lou Z, Wang L (2011). The role of FKBP5 in cancer aetiology and chemoresistance. Br J Cancer.

[7] van de Hoef DL, Bonner JM, Boulianne GL (2013). FKBP14 is an essential gene that regulates Presenilin protein levels and notch signaling in Drosophila. Development.

[8] Wang YQ, Zeng LZ, Xing D (2015). ROS-mediated enhanced transcription of CYP38 promotes the plant tolerance to high light stress by suppressing GTPase activation of PsbO2. Front Plant Sci.

[9] Singh K, Zouhar M, Mazakova J, Rysanek P (2014). Genome-wide identification of the Immunophilin gene family in *Leptosphaeria maculans*: a causal agent of blackleg disease in oilseed rape (*Brassica napus*). OMICS.

[10] Gollan PJ, Bhave M (2010). Genome-wide analysis of genes encoding FK506-binding proteins in rice. Plant Mol Biol.

[11] Rubin GM, Yandell MD, Wortman JR, Miklos GLG, Nelson CR, Hariharan IK (2000). Comparative genomics of the eukaryotes. Science.

[12] Efremov RG, Leitner A, Aebersold R, Raunser S (2015). Architecture and conformational switch mechanism of the ryanodine receptor. Nature.

[13] Boudko SP, Ishikawa Y, Nix J, Chapman MS, Bachinger HP (2014). Structure of human peptidyl-prolyl cis-trans isomerase FKBP22 containing two EF-hand motifs. Protein Sci.

[14] Larson A, Fair BJ, Pleiss JA (2016). Interconnections between RNA-processing pathways revealed by a sequencing-based genetic screen for pre-mRNA splicing mutants in fission yeast. G3 (Bethesda).

[15] Preall JB, Czech B, Guzzardo PM, Muerdter F, Hannon GJ (2012). Shutdown is a component of the Drosophila piRNA biogenesis machinery. RNA.

[16] Assimon VA, Southworth DR, Gestwicki JE (2015). Specific binding of Tetratricopeptide repeat proteins to heat shock protein 70 (Hsp70) and heat shock protein 90 (Hsp90) is regulated by affinity and phosphorylation. Biochemistry.

[17] Cao W, Konsolaki M (2011). FKBP immunophilins and Alzheimer’s disease: a chaperoned affair. J Biosci.

[18] Munn K, Steward R (2000). The shut-down gene of Drosophila melanogaster encodes a novel FK506-binding protein essential for the formation of germline cysts during oogenesis. Genetics.

[19] Favrin G, Bean DM, Bilsland E, Boyer H, Fischer BE, Russel S (2013). Identification of novel modifiers of ab toxicity by transcriptomic analysis in the fruitfly. Sci Rep.

[20] Theopold U, Zotto LD, Hultmark D (1995). FKBP39, a Drosophila member of a family of proteins that bind the immunosuppressive drug FK506. Gene.

[21] Zaffran S (2000). Molecular cloning and embryonic expression of dFKBP59, a novel Drosophila FK506-binding protein. Gene.

[22] Bryant Z, Subrahmanyan L, Tworoger M, LaTray L, Liu CR, Li MJ (1999). Characterization of differentially expressed genes in purified Drosophila follicle cells: toward a general strategy for cell type-specific developmental analysis. PNAS.

[23] Liang L, Haug JS, Seidel CW, Gibson MC (2014). Functional genomic analysis of the periodic transcriptome in the developing Drosophila wing. Dev Cell.

[24] Buffin E, Gho M (2010). Laser microdissection of sensory organ precursor cells of Drosophila Microchaetes. PLoS One.

[25] Wu S, Gui J, Yin X, Pan Q, Liu X, Chu L (2016). Transmembrane domain is crucial to the subcellular localization and function of Myc target 1. J Cell Mol Med.

[26] Orian A, Steensel BV, Delrow J, Bussemaker HJ, Li L, Sawado T (2003). Genomic binding by the Drosophila Myc, max, mad/Mnt transcription factor network. Genes Dev.

[27] Hardie RC, Juusola M (2015). Phototransduction in Drosophila. Curr Opin Neurobiol.

[28] Barbagallo B, Garrity PA (2015). Temperature sensation in Drosophila. Curr Opin Neurobiol.

[29] Tian Y, Hu W, Tong HW, Han JH (2012). Phototransduction in Drosophila. China Life Sci.

[30] Montell C (2005). TRP channels in Drosophila photoreceptor cells. J Physiol.

[31] Zhang X, Trebak M (2014). Transient receptor potential canonical 7: a diacylglycerol-activated non-selective cation channel. Handb Exp Pharmacol.

[32] Worley PF, Zeng W, Huang G, Kim JY, Shin DM, Kim MS (2007). Homer proteins in Ca^2+^ signaling by excitable and non-excitable cells. Cell Calcium.

[33] Zimmermann J, Latta L, Beck A, Leidinger P, Fecher-Trost C, Schlenstedt G (2014). Trans-activation response (TAR) RNA-binding protein 2 is a novel modulator of transient receptor potential canonical 4 (TRPC4) protein. J Biol Chem.

[34] Stuhrman K, Roseberry AG (2015). Neurotensin inhibits both dopamine- and GABA-mediated inhibition of ventral tegmental area dopamine neurons. J Neurophysiol.

[35] Suliman MA, Zhang ZX, Na H, Ribeiro ALL, Zhang Y, Niang B (2016). Niclosamide inhibits colon cancer progression through downregulation of the notch pathway and upregulation of the tumor suppressor miR-200 family. Int J Mol Med.

[36] Sethi N, Kang Y (2012). Notch signaling: mediator and therapeutic target of bone metastasis. BoneKEy Reports.

[37] Ross RJ, Weiner MM, Lin H (2014). PIWI proteins and PIWI-interacting RNAs in the soma. Nature.

[38] Khurana JS, Theurkauf W (2010). piRNAs, transposon silencing, and Drosophila germline development. J Cell Biol.

[39] Zuo L, Wang Z, Tan Y, Chen XG, Luo XG (2016). piRNAs and their functions in the brain. Int J Hum Genet.

[40] Yang Q, Lin J, Liu M, Li RH, Tian B, Zhang X (2016). Highly sensitive sequencing reveals dynamic modifications and activities of small RNAs in mouse oocytes and early embryos. Sci Adv.

[41] Honda T, Tomonaga K (2016). Endogenous non-retroviral RNA virus elements evidence a novel type of antiviral immunity. Mob Genet Elements.

[42] Pek JW, Patil VS, Kai T (2012). piRNA pathway and the potential processing site, the nuage, in the Drosophila germline. Develop Growth Differ.

[43] Olivieri D, Senti KA, Subramanian S, Sachidanandam R, Brennecke J (2012). The cochaperone shutdown defines a group of biogenesis factors essential for all piRNA populations in Drosophila. Mol Cell.

[44] Minakhina S, Changela N, Steward R (2014). Zfrp8/PDCD2 is required in ovarian stem cells and interacts with the piRNA pathway machinery. Development.

[45] Fagegaltier D, Falciatori I, Czech B, Castel S, Perrimon N, Simcox A (2016). Oncogenic transformation of *Drosophila* somatic cells induces a functional piRNA pathway. Genes Dev.

[46] Brennecke J, Aravin AA, Stark A, Dus M, Kellis M, Sachidanandam R (2007). Discrete small RNA-generating loci as master regulators of transposon activity in Drosophila. Cell.

[47] Kelleher ES (2016). Reexamining the P-element invasion of Drosophila melanogaster through the Lens of piRNA silencing. Genetics.

[48] Ishizu H, Siomi H, Siomi MC (2012). Biology of PIWI-interacting RNAs: new insights into biogenesis and function inside and outside of germlines. Genes Dev.

[49] Guzzardo PM, Muerdter F, Hannon GJ (2013). The piRNA pathway in flies: highlights and future directions. Curr Opin Genet Dev.

[50] Xiol J, Cora E, Koglgruber R, Chuma S, Subramanian S, Hosokawa M (2012). A role for Fkbp6 and the chaperone machinery in piRNA amplification and transposon silencing. Mol Cell.

[51] Sinclair D, Fillman SG, Webster MJ, Weickert CS (2013). Dysregulation of glucocorticoid receptor co-factors FKBP5, BAG1 and PTGES3 in prefrontal cortex in psychotic illness. Sci Rep.

[52] Ebong I-O, Beilsten-Edmands V, Patel NA, Morgner N, Robinson CV (2016). The interchange of immunophilins leads to parallel pathways and different intermediates in the assembly of Hsp90 glucocorticoid receptor complexes. Cell Discov.

[53] Salminen A, Ojala J, Kaarniranta K, Hiltunen M, Soininen H (2011). Hsp90 regulates tau pathology through co-chaperone complexes in Alzheimer's disease. Prog Neurobiol.

[54] Dhamad AE, Zhou Z, Zhou JH, Du YC (2016). Systematic proteomic identification of the heat shock proteins (Hsp) that interact with estrogen receptor alpha (ERα) and biochemical characterization of the ERα-Hsp70 interaction. PLoS One.

[55] Rotoli D, Morales M, Maeso MDC, García MDP, Morales A, Ávila J (2016). Expression and localization of the immunophilin FKBP51 in colorectal carcinomas and primary metastases, and alterations following oxaliplatin-based chemotherapy. Oncol Letter.

[56] Koren J, Jinwal UK, Davey Z, Kiray J, Arulselvam K, Dickey CA (2011). Bending tau into shape: the emerging role of peptidyl prolyl-isomerases in tauopathies. Mol Neurobiol.

[57] Sinars CR, Cheung-Flynn J, Rimerman RA, Scammell JG, Smith DF, Clardy J (2003). Structure of the large FK506-binding protein FKBP51, an Hsp90 binding protein and a component of steroid receptor complexes. PNAS.

[58] O'Leary IIIJC, Zhang B, Koren IIIJ, Blair L, Dickey CA (2013). The role of FKBP5 in mood disorders: action of FKBP5 on steroid hormone receptors leads to questions about its evolutionary importance. CNS Neurol Disord Drug Targets.

[59] Yao Y-L, Liang Y-C, Huang H-H, Yang W-M (2011). FKBPs in chromatin modification and cancer. Curr Opin Pharmacol.

[60] Romano S, Di Pace A, Sorrentino A, Bisogni R, Sivero L, Romano MF (2010). FK506 binding proteins as targets in anticancer therapy. Anti Cancer Agents Med Chem.

[61] Pei H, Li L, Fridley BL, Jenkins GD, Kalari KR, Lingle W (2009). FKBP51 affects Cancer cell response to chemotherapy by negatively regulating Akt. Cancer Cell.

[62] Yang H, Zhang Q-X, Pei D-S, Xu F, Li Y, Yu R-T (2015). FK506-binding protein 5 inhibits proliferation and stimulates apoptosis of glioma cells. Arch Med Sci.

[63] Chambraud B, Sardin E, Giustiniani J, Dounane O, Schumacher M, Goedert M (2010). A role for FKBP52 in tau protein function. PNAS.

[64] Sanokawa-Akakura R, Cao W, Allan K, Patel K, Ganesh A, Heiman G (2010). Control of Alzheimer's amyloid Beta toxicity by the high molecular weight Immunophilin FKBP52 and copper homeostasis in Drosophila. PLoS One.

[65] Blair LJ, Baker JD, Sabbagh JJ, Dickey CA (2015). The emerging role of peptidyl-prolyl isomerase chaperones in tau oligomerization, amyloid processing and Alzheimer's disease. J Neurochem.

[66] Dakson A, Yokota O, Esiri M, Bigio EH, Horan M, Pendleton N (2011). Granular expression of prolyl-peptidyl isomerase PIN1 is a constant and specific feature of Alzheimer’s disease pathology and is independent of tau, Aβ and TDP-43 pathology. Acta Neuropathol.

[67] Fontaine SN, Sabbagh JJ, Baker J, Martinez-Licha CR, Darling A, Dickey CA (2015). Cellular factors modulating the mechanism of tau protein aggregation. Cell Mol Life Sci.

[68] Brai E, Raio NA, Alberi L (2016). Notch1 hallmarks fibrillary depositions in sporadic Alzheimer’s disease. Acta Neuropathol Commun.

[69] Xu X, Su B, Barndt RJ, Chen H, Xin H, Yan G, Chen L, Cheng D, Heitman J, Zhuang Y, Fleischer S, Shou W (2002). FKBP12 is the only FK506 binding protein mediating T-cell inhibition by the immunosuppressant FK506. Transplantation.

[70] Maruyama M, Li BY, Chen H, Xu X, Song LS, Guatimosim S, Zhu W, Yong W, Zhang W, Bu G, Lin SF, Fishbein MC, Lederer WJ, Schild JH, Field LJ, Rubart M, Chen PS, Shou W (2011). FKBP12 is a critical regulator of the heart rhythm and the cardiac voltage-gated sodium current in mice. Circ Res.

[71] des Georges A, Clarke OB, Zalk R, Yuan Q, Condon KJ, Grassucci RA, Hendrickson WA, Marks AR, Frank J (2016). Structural Basis for Gating and Activation of RyR1. Cell.

[72] Shou W, Aghdasi B, Armstrong DL, Guo Q, Bao S, Charng MJ, Mathews LM, Schneider MD, Hamilton SL, Matzuk MM (1998). Cardiac defects and altered ryanodine receptor function in mice lacking FKBP12. Nature.

[73] Xin HB, Senbonmatsu T, Cheng DS, Wang YX, Copello JA, Ji GJ, Collier ML, Deng KY, Jeyakumar LH, Magnuson MA, Inagami T, Kotlikoff MI, Fleischer S (2002). Oestrogen protects FKBP12.6 null mice from cardiac hypertrophy. Nature.

[74] Zalk R, Clarke OB, des Georges A, Grassucci RA, Reiken S, Mancia F, Hendrickson WA, Frank J, Marks AR. Structure of a mammalian ryanodine receptor. Nature 2015;517(7532):44–49. doi:10.1038/nature13950. 10.1038/nature13950 PMC4300236 25470061

[75] Prakash A, Rajan S, Yoon HS (2016). Crystal structure of the FK506 binding domain of human FKBP25 in complex with FK506. Protein Sci.

[76] Prakash A, Shin J, Rajan S, Yoon HS (2016). Structural basis of nucleic acid recognition by FK506-binding protein 25 (FKBP25), a nuclear immunophilin. Nucleic Acids Res.

[77] Edlich-Muth C, Artero J-B, Callow P, Przewloka MR, Watson AA, Wei Zhang W (2015). Et. The Pentameric Nucleoplasmin fold is present in Drosophila FKBP39 and a large number of chromatin-related proteins. J Mol Biol.

[78] Przewloka MR, Zhang W, Costa P, Archambault V, D'Avino PP, Lilley KS (2007). Molecular analysis of core kinetochore composition and assembly in Drosophila melanogaster. PLoS One.

[79] Hughes JR, Meireles AM, Fisher KH, Garcia A, Antrobus PR, Wainman A (2008). A microtubule interactome: complexes with roles in cell cycle and mitosis. PLoS Biol.

[80] Kozłowska M, Tarczewska A, Jakób M, Bystranowska D, Taube M, Kozak M (2016). Nucleoplasmin-like domain of FKBP39 from Drosophila melanogaster forms a tetramer with partly disordered tentacle-like C-terminal segments. Sci Rep.

[81] Li Y, Zhang Z, Robinson GE, Palli SR (2007). Identification and characterization of a juvenile hormone response element and its binding proteins. J Biol Chem.

[82] Kozłowska M, Tarczewska A, Jakób M, Szpotkowski K, Wojtas M, Rymarczyk G (2014). Calponin-like Chd64 is partly disordered. PLoS One.

[83] Xu X, Bhat MB, Nishi M, Takeshima H, Ma J (2000). Molecular cloning of cDNA encoding a Drosophila ryanodine receptor and functional studies of the carboxyl-terminal calcium Release Channel. Biophys J.

